# A monomolecular platform with varying gated photochromism†

**DOI:** 10.1039/d0ra08214g

**Published:** 2020-11-19

**Authors:** Yuezheng Li, Xuanying Chen, Taoyu Weng, Jufang Yang, Chunrui Zhao, Bin Wu, Man Zhang, Liangliang Zhu, Qi Zou

**Affiliations:** Shanghai Key Laboratory of Materials Protection and Advanced Materials in Electric Power, Shanghai University of Electric Power Shanghai 200090 China qzou@shiep.edu.cn; State Key Laboratory of Molecular Engineering of Polymers, Department of Macromolecular Science, Fudan University Shanghai 200438 China

## Abstract

In the development of modern high-performance photoelectric materials, the gated photochromic materials have attracted wide attention. However, the integration of varying signal regulations into gated photochromism to construct efficient photochromic materials is still an urgent necessity. Herein, we designed and synthesized a new gated photoswitching DTEP based on a Schiff base with a diarylethene core. The photochromic properties of compound DTEP can be regulated to different degrees by multiple stimuli, including UV/visible light, Cu^2+^ and Ni^2+^. The compound DTEP showed different response abilities to Cu^2+^ and Ni^2+^, due to the diverse complexation modes between DTEP and Cu^2+^ as well as Ni^2+^. The photochromic properties of compound DTEP could be inhibited completely by the introduction of Cu^2+^ to form a 1 : 1 complexation, while the weak gated photochromism could be found from the DTEP–Ni^2+^ complex in a 1 : 2 stoichiometry. Relying on such varying degrees of gated photochromic properties, a new molecular logic circuit was constructed to undertake complicated logical operations.

## Abstract 2

A strategy to realize varying degrees of gated photochromic properties by coordinating with different metal ions within one unimolecular system was devised to achieve the construction of a logic circuit for multi-functional molecular switching.

## Graphical abstract

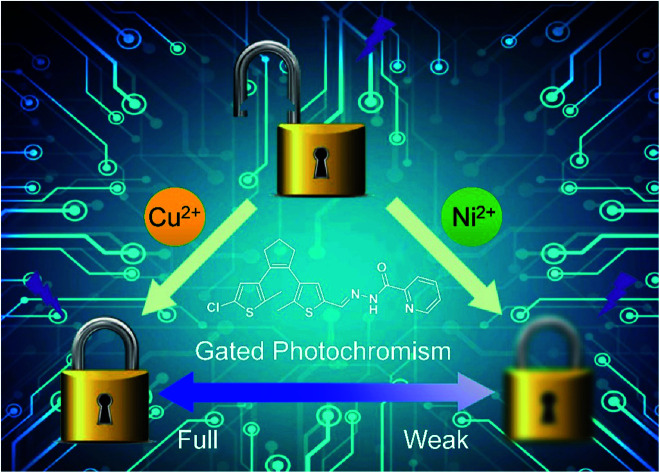

## 1. Introduction

Photochromic compounds, as a kind of molecular switch, which can undergo reversible photoisomerization between two stable forms, making an important contribution for the development of composite materials by achieving automatic regulation over their properties under certain external stimuli.^1–4^ Diarylethene derivatives are considered to be an outstanding representative of this kind of photoswitching materials, due to their good thermal stability, high fatigue resistance and short photoresponse time, as well as high ring-closure and ring-open quantum yields. Such unique photochromic properties and reversible molecular structures bring about their wide applications in smart materials, chemical probes, optical switches and memories, *etc.*^5–11^

In recent years, diarylethene derivatives with gated photochromic properties have gradually become a hot topic in the field of photoelectric materials.^12–16^ These gated photochromic properties endow diarylethene derivatives with non-destructive readout, which can efficiently inhibit the proportional photons absorbed by molecules within normal photochromic reactions, further gaining a nonlinear response that can be applied in optoelectronic devices, for instance, optical data storages, chemical sensors and photoresponsive self-assemblies.^9,17–21^ So far, several strategies have been adopted to achieve the gated photochemical reactions of diarylethene derivatives, such as fixing two aryl rings in a parallel conformation,^22^ altering the structure of ethylene bridge based on chemical reaction,^23–25^ embellishing substituent groups of the aryl rings,^26,27^ inhibiting ring–opening reaction by decreasing temperature,^28^ multi-photon-gated photochemical reaction,^29^ quenching of photo-reactive excited state,^30^ modifying reactive surface within self-assembled monolayer or polymer film,^31^ and forming intermolecular hydrogen bonds to control the conformation.^22,32^

**Scheme 1:**
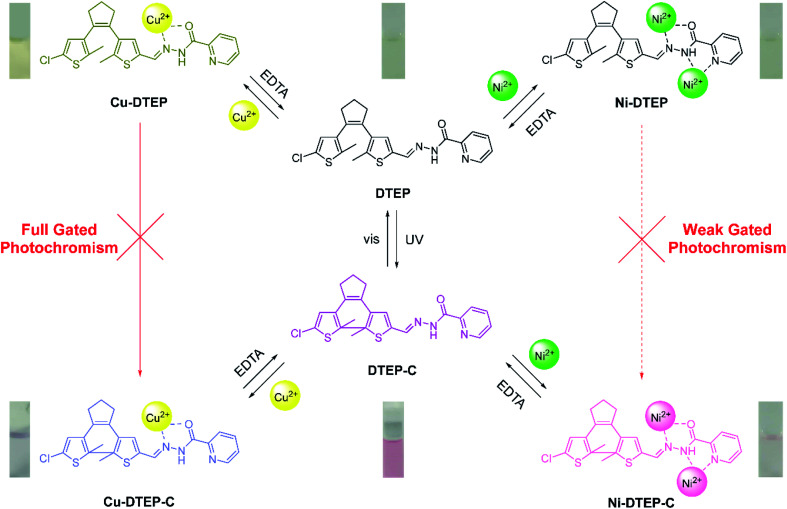
Responses of compound DTEP to UV, visible light, Cu^2+^ and Ni^2+^ stimuli. Inset: the corresponding color changes under diverse stimuli.

However, it is still a great challenge to integrate gated photoreactivity controlled in difference degrees by multiple stimuli into a monomolecular platform in order to serve as molecular logic devices and specific stimuli-gated molecular switches.^7^ In another hand, compared with chemical reactions and heating, the photoreactivity gated by coordination or complexation is superior, on account of the simple and reversible operation modes between molecules and economical complexation agents by the coordination and dissociation processes.^13–16^ Herein, we designed and synthesized a Schiff base DTEP (see Scheme 1) featured with a diarylethene core and 2-pyridine formhydrazide subunit to respond to Cu^2+^ and Ni^2+^ with high selectivity. Compound DTEP exhibited the typical photoisomerization reaction upon alternative irradiation with UV and visible light. The diverse gated photoreactivity of compound DTEP could be found in the presence of Cu^2+^ and Ni^2+^, due to the different complexation patterns between DTEP and Cu^2+^ as well as Ni^2+^. Based on these photochromic properties, we designed a multi-stimuli-responding molecular circuit with complicated logic operations. The synthetic route of compound DTEP is shown in Scheme S1.† The structure of compound DTEP was well confirmed by ^1^H NMR, ^13^C NMR, and HRMS (Fig. S12–S14,† see the Experimental section and ESI† for details).

## 2. Experimental section

### 2.1 Materials and instrumentations

1-(5-Chloro-2-methyl-3-thienyl)-2-(5-formyl-2-methyl-3-thienyl)cyclopentene was prepared and purified according to literature procedures.^33^ All other reagents were of analytical purity and used without further treatment. Thin-layer chromatography (TLC) analyses were performed on silica-gel plates, and flash chromatography was conducted by using silica-gel column packages purchased from Qing-dao Haiyang Chemical Company, China.

^1^H NMR and ^13^C NMR spectra in CDCl_3_ were recorded on Brucker AM-400 spectrometers with tetramethylsilane (TMS) as the internal standard. High-resolution mass spectrometry (HR-MS) were measured by Matrix Assisted Laser Desorption Ionization-Time of Flight/Time of Flight Mass Spectrometer (5800). The melting point was measured by micro-melting point instrument SGWX-4. UV-vis absorption spectra were recorded on a Shimadzu 1800 spectrophotometer, while the fluorescent emission spectra were taken with a Shimadzu RF-5301 PC; both spectrophotometers were standardized.

### 2.2 Synthesis of compound DTEP

The mixture of 1-(5-chloro-2-methyl-3-thienyl)-2-(5-formyl-2-methyl-3-thienyl)cyclopentene (1.20 g, 3.72 mmol) and 2-pyridine-carbo-xyhydrazide (0.76 g, 5.58 mmol) in ethanol (10.0 ml) was refluxed for 12 h under nitrogen atmosphere. After cooling to room temperature, the mixture was filtered, and the residue was washed with cold ethanol to give the compound DTEP (1.15 g, 70%) as a yellow powder. ^1^H NMR (400 MHz, CDCl_3_, 298 K) *δ* (ppm): 1.84 (s, 3H, –CH_3_), 2.01–2.08 (m, 5H, –CH_3_ and –CH_2_–), 2.71–2.80 (m, 4H, –CH_2_–), 6.60 (s, 1H, thiophene-H), 7.00 (s, 1H, thiophene-H), 7.48 (t, *J* = 6.0 Hz, 1H, pyridine-H), 7.90 (t, *J* = 5.0 Hz, 1H, pyridine-H), 8.28 (d, *J* = 8.0 Hz, 1H, pyridine-H), 8.49 (s, 1H, –CH=N–), 8.57 (d, *J* = 4.0 Hz, 1H, pyridine-H), 10.86 (s, 1H, –NH–N–). ^13^C NMR (100 MHz, CDCl_3_) *δ* (ppm): 14.14, 14.91, 22.88, 38.26, 38.34, 120.79, 122.19, 122.75, 125.24, 126.64, 133.33, 136.17, 137.62, 139.47, 140.26, 144.05, 146.41, 148.05, 149.19, 155.49, 159.95, 166.88. HRMS (MALDI-TOF, *m*/*z*): [M + H]^+^ calcd for C_22_H_20_ClN_3_OS_2_, 442.0736; found, 442.0648. Mp: 116 °C.

## 3. Results and discussion

### 3.1 Photophysical properties

**Fig. 1:**
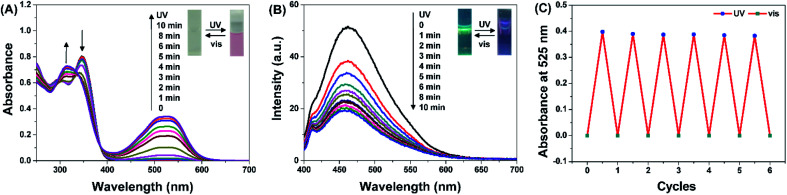
The UV-vis absorption (A) and fluorescence spectral changes (B) of compound DTEP (10 μM) in MeOH solution with the irradiation of 365 nm light at 25 °C. *λ*_ex_ = 365 nm, slits: 5 nm/5 nm. Inset: the corresponding photographic images upon irradiation with UV and visible light. (C) Fatigue resistance of compound DTEP irradiated by UV (365 nm) and visible light (*λ* > 500 nm) alternately.

The UV-vis absorption and fluorescent spectra of compound DTEP were measured in methanol (MeOH) solution at room temperature, respectively. As displayed in Fig. 1A, a significant absorption peak appeared at 525 nm with the irradiation of 365 nm light, and the absorbance band centered at 525 nm gradually increased with the extension of irradiation time. In the meantime, the absorption band at 346 nm disappeared while a new absorption peak appeared at 314 nm. The photostationary state was reached after UV irradiation for 10 min along with the change of the colorless solution to pink (Scheme 1). As shown in Fig. 1B, the emission at 460 nm belonging to compound DTEP was quenched to ∼35% of the initial value after irradiation with 365 nm light for 10 min. Such spectral and color changes are attributed to the formation of ring-closed isomer DTEP-C as shown in Scheme 1. If the solution assigned to the photostationary state of compound DTEP was exposed to visible light (>500 nm), the UV-vis absorption and fluorescent spectra as well as color could recover. Therefore, it is concluded that compound DTEP possesses typical photochromic properties under the alternate irradiation of UV and visible light.

The fatigue resistance of DTEP in methanol was studied by the alternating irradiation of UV and visible light. As seen from Fig. 1C, the absorbance at 525 nm did not show distinguished change after six cycles of alternant UV/visible light irradiation, suggesting the excellent fatigue resistance of compound DTEP. In addition, the thermal stability of compound DTEP in methanol was tested at 338 K for 48 h in the dark. As shown in Fig. S1,† the resulting solution still exhibited the similar photochromic behaviors in comparison with the original solution, indicating that compound DTEP possessed the good thermal stability.

### 3.2 Responses to Cu^2+^ and Ni^2+^

**Fig. 2:**
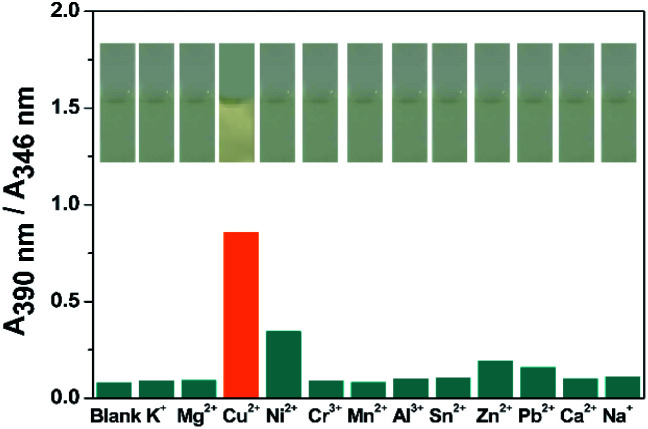
The absorbance ratio (*A*_390 nm_/*A*_346 nm_) of compound DTEP (10 μM) upon the titration of various metal ions (40 μM) in MeOH solution at 25 °C and corresponding color changes (inset).

The sensing ability of compound DTEP to common metal ions were measured in methanol solution at room temperature, including K^+^, Mg^2+^, Cr^3+^, Mn^2+^, Al^3+^, Sn^2+^, Zn^2+^, Pb^2+^, Ca^2+^, and Na^+^. Accordingly, the ratio of the absorbance at 390 nm and 346 nm (*A*_390 nm_/*A*_346 nm_) with addition of 4.0 equiv. of metal ions is considered as the sensing index and summarized in Fig. 2, respectively. It is found that Cu^2+^ and Ni^2+^ could efficiently result in the significant change on *A*_390 nm_/*A*_346 nm_ and obvious color change was observed for Cu^2+^ (Fig. 2, inset), showing the high selectivity of compound DTEP to Cu^2+^ and Ni^2+^, respectively. In the meanwhile, to exclude the disturbance of anions, we measured the UV-vis absorption spectra of compound DTEP and its photostationary state with Cl^−^, I^−^, F^−^, Br^−^, CN^−^, ClO_4_^−^, BF_4_^−^, PF_6_^−^ in methanol solution, respectively. It can be seen from Fig. S2† that no obvious spectral response of the compound DTEP to various anions was observed, suggesting that compound DTEP can effectively respond to Cu^2+^ and Ni^2+^ in the more complex solution environment.

Therefore, we focused on investigating the absorption and fluorescence spectral responses of compound DTEP to Cu^2+^ and Ni^2+^ under the same conditions, respectively. As shown in Fig. S3A,† the absorbance at 346 nm gradually decreased along with a new absorption band appeared at 390 nm through the continuous addition of Cu^2+^ (0–4.0 equiv.), and at the same time, the fluorescence was quenched completely (Fig. S3B†). Fig. S4† suggests that the saturated amount of Ni^2+^ (4.0 equiv.) to the methanol solution of compound DTEP could induce the bathochromic shift of the absorption band centered at 346 nm to 355 nm accompanied by the quenching of fluorescence to a certain extension. The gradual changes of the absorption and fluorescent spectra are ascribed to the formation of the complexes between compound DTEP and Cu^2+^ as well as Ni^2+^ in 1 : 1 and 1 : 2 stoichiometry, respectively, which is confirmed by the MS (MALDI-TOF) analysis (the details as shown in Fig. S5†).

Due to the paramagnetic effect of Cu^2+^, we could not utilize ^1^H NMR titration experiments to verify the binding mode of compound DTEP with Cu^2+^, however, based on our reports,^13–16^ we assume that N atom within imide group and O atom within amide group take part in forming complex together as displayed in Scheme 1. As shown in Fig. S6,†^1^H NMR titration of Ni^2+^ to compound DTEP shows that N atom in imide group plays a key role in the coordination, in combination with previous report,^34^ it is reasonable to infer that N atoms in imide, amide and pyridine groups, and O atom in amide group coordinate to Ni^2+^ with a 1 : 2 binding mode as depicted in Scheme 1.

We also found that compound DTEP in the photostationary state could selectively respond to Cu^2+^ and Ni^2+^ with different absorption and color changes (Fig. S7†), respectively, suggesting that compound DTEP in methanol solution could serve as a photo-controlled chemosensor to detect Cu^2+^ and Ni^2+^ with high selectivity. Moreover, the original UV-vis absorption and fluorescent spectra could be recovered by extracting Cu^2+^ and Ni^2+^ from the corresponding complexes and their photostationary state solutions using ethylenediami-netetraacetae (EDTA), showing a handy manner to tune the photochromic properties of compound DTEP by the straightforward processes of complexation and dissociation.

### 3.3 Gated photoreactivity

**Fig. 3:**
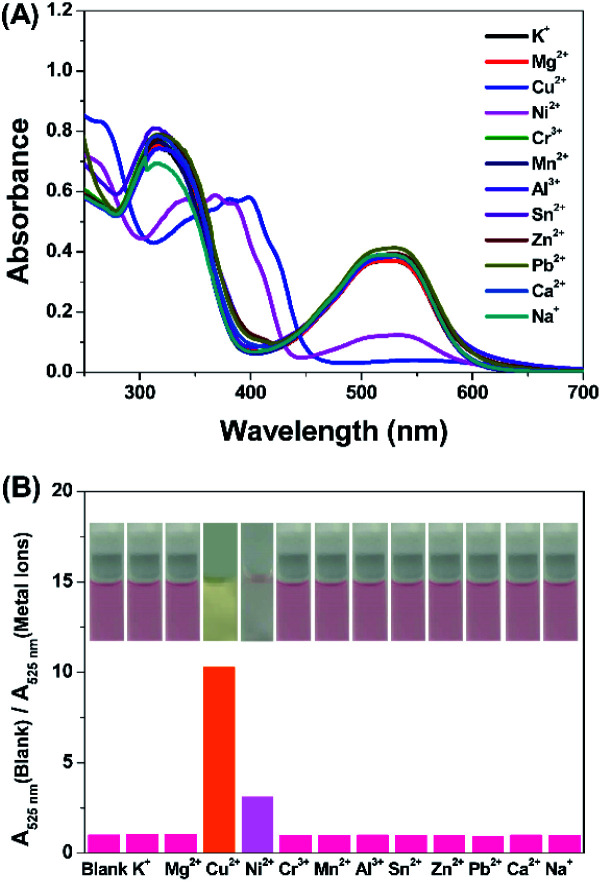
(A) UV-vis absorption spectra of compound DTEP (10 μM) with various metal ions (40 μM) in MeOH solution after irradiation with 365 nm light. (B) The ratio of absorbance at 525 nm of compound DTEP (10 μM) without and with various metal ions (40 μM) in MeOH solution after irradiation with 365 nm light at 25 °C. Inset: the corresponding photographic images of compound DTEP with various metal ions (40 μM) after irradiation with 365 nm light.

**Fig. 4:**
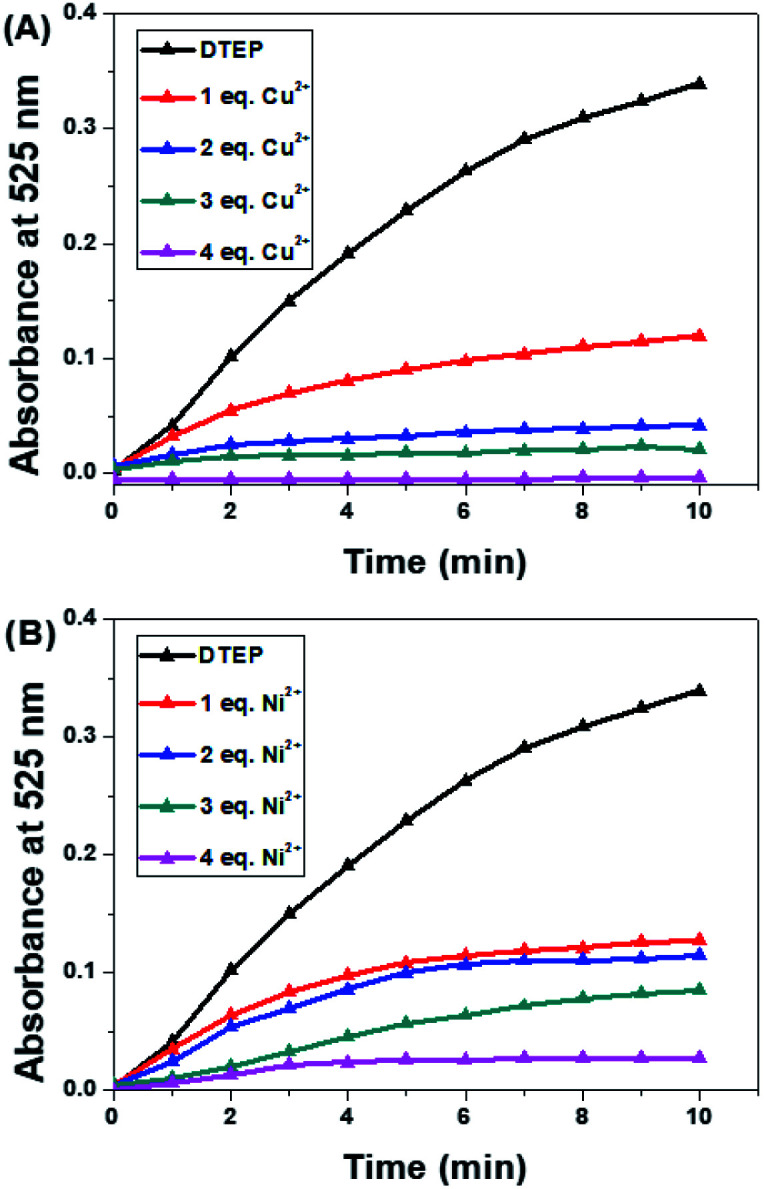
Absorbance at 525 nm of compound DTEP (10 μM) with different amounts of Cu^2+^ (A) and Ni^2+^ (B) over irradiation time in MeOH solutions: none; 1 equiv.; 2 equiv.; 3 equiv. and 4 equiv.

The photoactivity of compound DTEP in methanol solution was fully investigated in the presence of metal ions. In this study, the methanol solution of compound DTEP with various metal ions was irradiated by 365 nm UV light for 10 min to ensure to reach the photostationary state and the resulting absorption spectra were summarized in Fig. 3A. Additionally, the ratio of absorbance at 525 nm (*A*_525 nm_ (blank)/*A*_525 nm_ (metal ions)) of compound DTEP in methanol solution without and with various metal ions after irradiation with 365 nm light was presented in Fig. 3B. As shown in Fig. 3, Cu^2+^ and Ni^2+^ could uniquely suppress the photoresponsive ability of compound DTEP. It is worthy that the introduction of Cu^2+^ could inhibit the photoreactivity of compound DTEP in methanol solution completely. As seen from Fig. S8,† the absorption spectrum of compound DTEP with 4 equiv. of Cu^2+^ was still consistent even with the irradiation of 365 nm light for 10 min. Additionally, complex Cu-DTEP could not take place the efficient photochromic transformation with any wavelength of light, indicating the photo-inactivity of the complex of DTEP with Cu^2+^. Previous studies on the gated photoreactivity have rationalized that the interaction between DTEP and Cu^2+^ completely impedes the electrocyclization reaction among two activated carbon atoms assigned to thiophene moieties.^14,16^ In order to further investigate the effect of different amounts of Cu^2+^ on the gated photoreactivity of compound DTEP, the dependence of absorbance at 525 nm with irradiation time is plotted in Fig. 4A. It is concluded that the efficiency of the photo–cyclization reaction is reduced and finally lost by the persistent addition of Cu^2+^ till to 4.0 equiv. to result in forming the complex Cu-DTEP completely.

In order to gain insight into photochromism gated by Ni^2+^, different amounts of Ni^2+^ (1.0, 2.0, 3.0 and 4.0 equiv.) were added to the methanol solution of compound DTEP, the producing complex solutions were ensured to reach the photostationary state through irradiation with 365 nm light for 10 min, respectively. Fig. 4B and S9† demonstrates that the absorption band at 525 nm weakened gradually under UV light irradiation as the increase of Ni^2+^ in solution. However, the photoreactivity could not disappear even further increase of Ni^2+^. It is a new type of regulation concept that photoreactivity is gated by complexation with metal ions in part. To the best of our knowledge, it is the first report on the unique weak gated effect of metal ions responded to diarylethene derivatives. Such a weak gated photochromism should be attributed to the weaker electrocyclization ability between two activated carbon atoms resulted from the different binding mode of compound DTEP with Ni^2+^. In other words, we employed a powerful tool to modulate the gated photoreactivity by the formation of various complexes upon addition of with specific metal ions.

Besides, we further explored the impact of water in methanol solution to the gated photoreactivity. As shown in Fig. S10,† the similar photochromic properties of compound DTEP could be observed in methanol–water solutions with different ratio (9 : 1, 7 : 3, and 5 : 5, v/v) water, suggesting that water in methanol solution was not able to activate the gated photoreactivity of compound DTEP independently. We further carried out UV light irradiation experiments of complex Cu-DTEP and Ni-DTEP in methanol–water (5 : 5, v/v) solution, respectively. It can be seen from Fig. S11† that the photoreactivity of compound DTEP was hindered or suppressed by adding Cu^2+^ or Ni^2+^ likewise that in methanol solutions. These results suggest that the water effect can be ignored during the gated photochromic behaviors of compound DTEP, further to the benefit of extending the application scope of compound DTEP.

Interestingly, such varying degrees of gated photochromism can be regulated further by the complexation and dissociation of compound DTEP with Cu^2+^ and Ni^2+^. By adding Cu^2+^ or Ni^2+^ into the methanol solution of compound DTEP, the “locking” process of the photoreactivity occurred, and then EDTA could give birth to the “unlocking” process by extraction of metal ions from the corresponding complexes, showing a restoration of the photoreactivity.

## 4. Application as a logic circuit

**Fig. 5:**
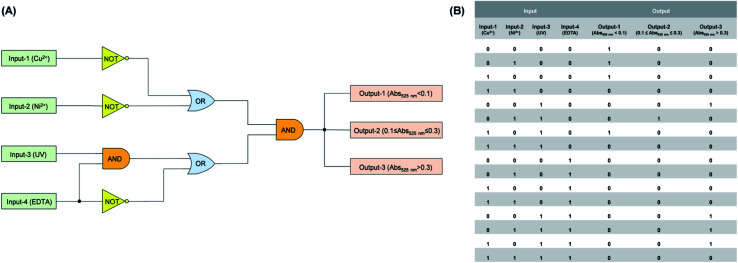
(A) A logic circuit with four inputs and three outputs: input-1 (Cu^2+^ addition), input-2 (Ni^2+^ addition), input-3 (UV light irradiation), input-4 (EDTA addition), output-1 (absorbance at 525 nm < 0.1), output-2 (0.1 ≤ absorbance at 525 nm ≤ 0.3), output-3 (absorbance at 525 nm > 0.3). (B) Truth table for the molecular logic circuit.

As one type of logic devices, logic circuit with multiple input and output signals has been designed delicately and shows great potential in logic operations.^35–39^ As mentioned above, varying degrees of gating photochromic properties were realized by the introduction of Cu^2+^ and Ni^2+^ to the methanol solution of compound DTEP. On this basis, a smart logic circuit (Fig. 5A) was constructed to conduct complex logic operations with four input signals (input-1: Cu^2+^ addition, input-2: Ni^2+^ addition, input-3: UV light irradiation, input-4: EDTA addition) and three output signals (output-1: absorbance at 525 nm < 0.1; output-2: 0.1 ≤ absorbance at 525 nm ≤ 0.3; output-3: absorbance at 525 nm > 0.3). As seen from Fig. 5B, Booleans “0” and “1” mean the absence and presence of stimuli (*e.g.* metal ions, light and EDTA) corresponding to four input signals. With regard to output-1 signal, Boolean “1” means that the absorbance at 525 nm is less than 0.1, indicating the complete inhibition of the photochromic behavior of compound DTEP; for output-2 signal, Boolean “1” means the absorbance at 525 nm is between 0.1 and 0.3, indicating the weak gated photochromism of compound DTEP; for output-3 signal, the absorbance at 525 nm is more than 0.3, demonstrating the photochromism feasible. Otherwise, Boolean is “0”. The diversity of input and output signals enables the simple system to undertake the more complicated logical operations, while the simple and efficient properties of compound DTEP are preserved. Such logic circuit with the multi-signal channels based on unimolecular platform could be a promising candidate for the smart logic materials.

## 5. Conclusion

In this paper, a novel diarylethene derivative DTEP featured with the Schiff base group was developed. Compound DTEP exhibited remarkable photochromic properties and switchable fluorescence by alternating UV and visible light irradiation. And it can serve as a photo-controlled probe for detecting Cu^2+^ and Ni^2+^ by absorption and fluorescence changes with high selectivity. More importantly, the photo-reactivity of compound DTEP can be suppressed in different degrees by the addition of Cu^2+^ and Ni^2+^ to its methanol solutions. The full gated photochromic properties by Cu^2+^ are observed, which is stemmed from the formation of a 1 : 1 binding complex with the strong interaction between compound DTEP and Cu^2+^. In another hand, a novel type of weak gated photochromism is conceived by the infirm DTEP–Ni^2+^ interaction according to the different coordination manner of compound DTEP with Ni^2+^ in 1 : 2 stoichiometry. Relying on the varying gated photoreactivity of compound DTEP by forming the different complexes with Cu^2+^ and Ni^2+^, a molecular logic circuit with four inputs and three outputs was designed to perform intricate logic operations. Such a unimolecular platform with varying gated photochromism is of great significance for the development of new novel molecular switches and smart materials.

## Conflicts of interest

There are no conflicts to declare.

## Supplementary Material

RA-010-D0RA08214G-s001
