# Supplementary material for: A monomolecular platform with varying gated photochromism
Source: RSC Adv. 2020 Nov 19;10(69):42194–9. doi: 10.1039/d0ra08214g (PMC9057850; doi:10.1039/d0ra08214g)
Supplement: RA-010-D0RA08214G-s001 [file RA-010-D0RA08214G-s001.pdf]

Electronic Supplementary Material (ESI) for RSC Advances.
This journal is © The Royal Society of Chemistry 2020

Electronic Supplementary Information

A molecular switch with varying gated photochromism

Yuezheng Li,[a] Xuanying Chen,[a] Taoyu Weng,[a] Jufang Yang,[a] Chunrui Zhao,[a] Bin Wu,[b] Man Zhang,[b] Liangliang Zhu,[b] and Qi Zou[a]*

[a] *Shanghai Key Laboratory of Materials Protection and Advanced Materials in Electric Power*

Shanghai University of Electric Power, Shanghai 200090, China

* *E-mail: qzou@shiep.edu.cn (Q. Zou)*

[b] *State Key Laboratory of Molecular Engineering of Polymers*

Department of Macromolecular Science

Fudan University, Shanghai 200438, China

Supplementary scheme and figures

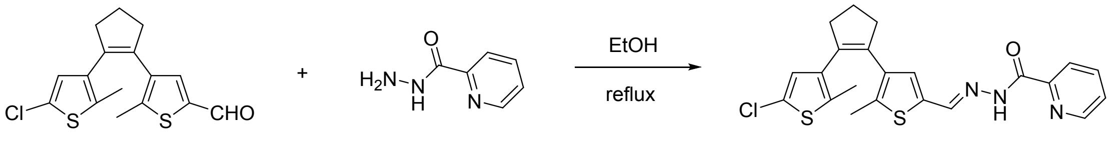

DTEP

Scheme S1. Synthesis route of compound **DTEP**.

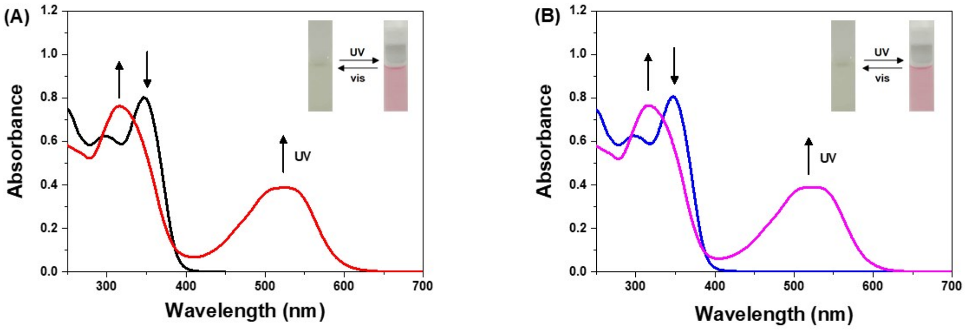

Figure S1. The UV-vis absorption spectral changes of compound **DTEP** (10 μM) upon alternative irradiation with UV and visible light in MeOH solution at 25° C: **(A)** the original solution; **(B)** the solution heated at 338 K for 48 h.

The irradiated solution still exhibited the similar photochromic behaviors in comparison with the original solution, indicating that compound **DTEP** possessed the good thermal stability.

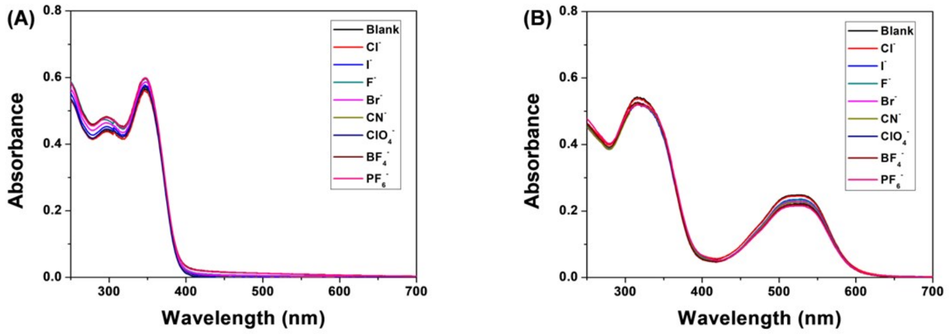

Figure S2. The UV-vis absorption changes of **(A)** compound **DTEP** (10 μM) and **(B)** its photostationary state with various anions (40 μM) in MeOH solution at 25 °C.

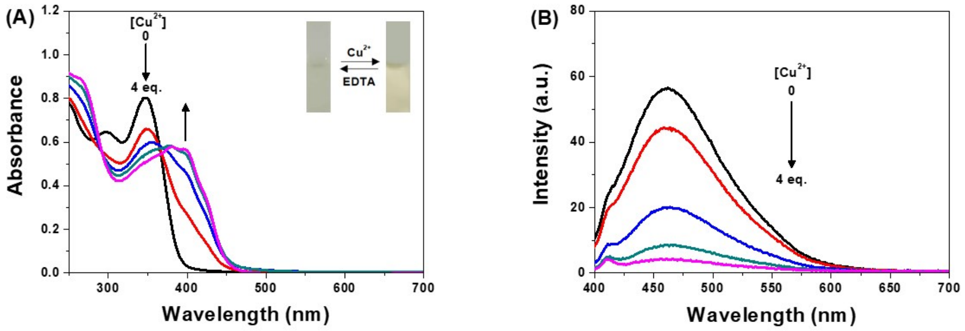

Figure S3. UV-vis absorption **(A)** and fluorescence changes **(B)** of compound **DTEP** (10 μM) with the addition of Cu^{2+} (0-40 μM) in MeOH solution at 25° C. λ_{ex} = 365 nm, slits: 5 nm/ 5 nm. Inset: the corresponding photographic images upon irradiation with UV and visible light.

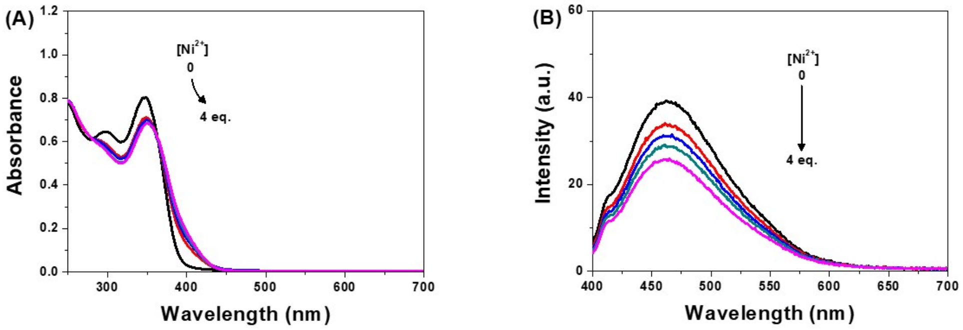

Figure S4. UV-vis absorption **(A)** and fluorescence changes **(B)** of compound **DTEP** (10 μM) with the addition of Ni^{2+} (0-40 μM) in MeOH solution at 25° C. λ_{ex} = 365 nm, slits: 5 nm/ 5 nm.

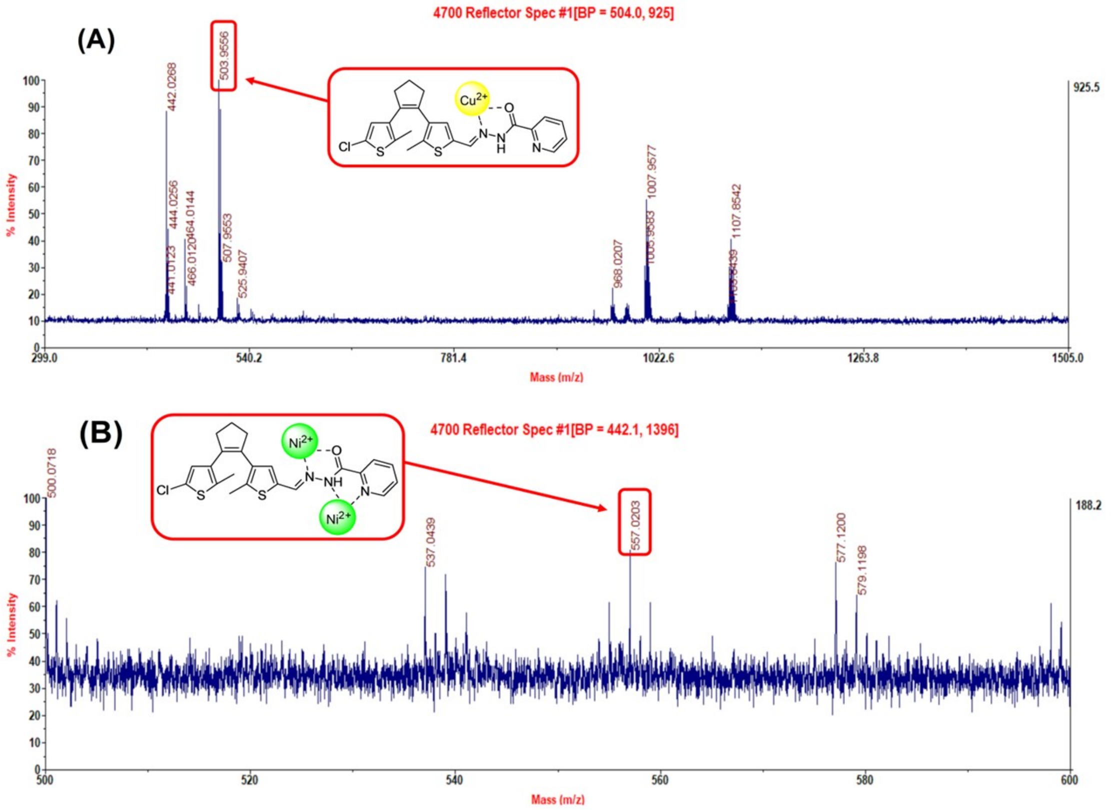

Figure S5. MS (MALDI-TOF) of compound **DTEP** (10 μM) with Cu^{2+} **(A)** and Ni^{2+} **(B)** ions (40 μM) in MeOH solution at 25 ℃.

When 4.0 equiv. of Cu^{2+} ions was added to the **DTEP** solution, a new peak appeared at *m/z* 503.9556 (Figure S4A) that was attributed to the [**DTEP**+Cu^{2+}] adduct (the calculated value was 504.0121), suggesting the complex of **DTEP** and Cu^{2+} ions with a binding stoichiometry of 1:1.

When 4.0 equiv. of Ni^{2+} ions was added to the **DTEP** solution, a new peak appeared at *m/z* 557.0203 (Figure S4B) that was attributed to the [Ni^{2+}+**DTEP**+Ni^{2+}] adduct (the calculated value was 556.9421), suggesting the complex of **DTEP** and Ni^{2+} ions with a binding stoichiometry of 1:2.

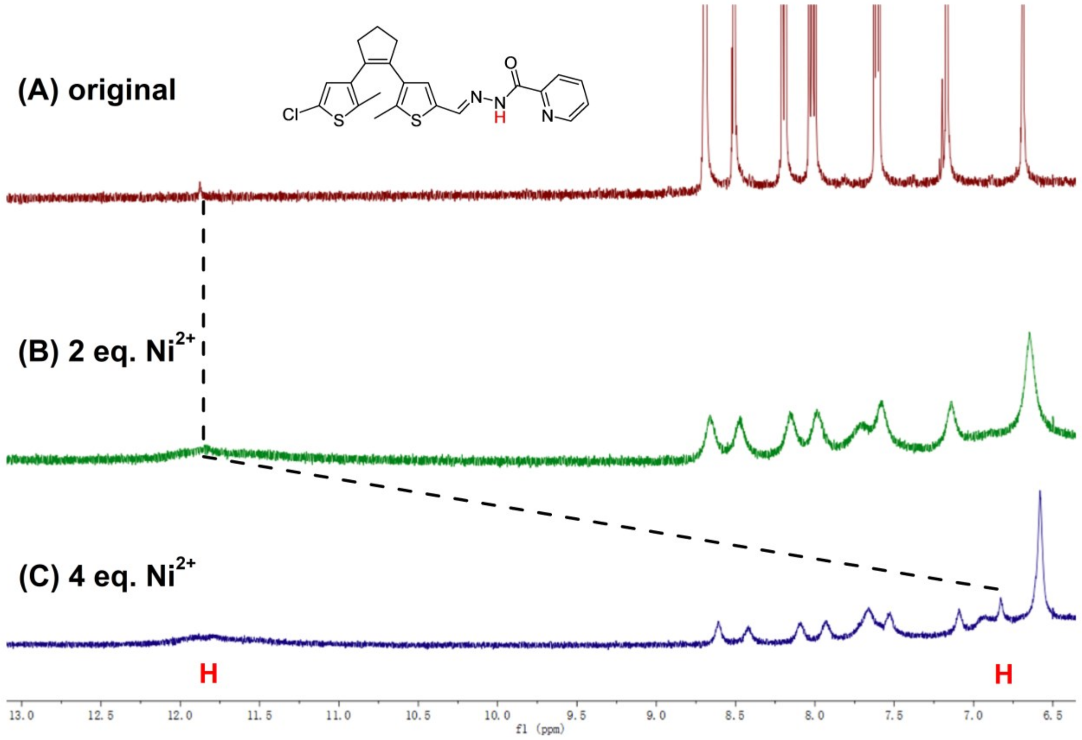

Figure S6. ^{1}H NMR titration of compound **DTEP** (10 μM) with Ni^{2+} ions (0-40 μM) in CD_3OD solution at 25 °C.

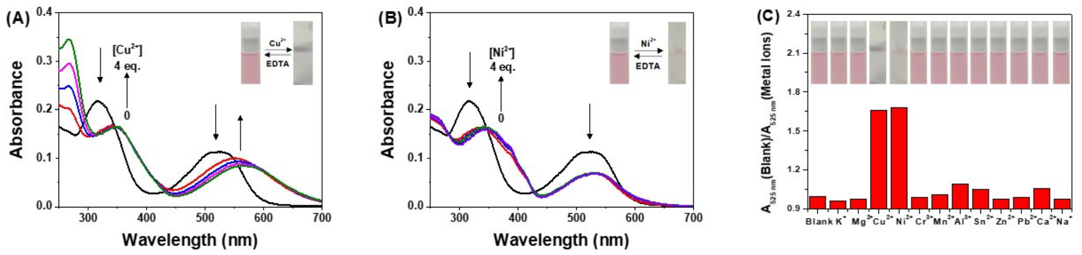

Figure S7. The UV-vis absorption changes of compound **DTEP** (10 μM) in the photostationary state upon the titration of **(A)** Cu^{2+} (0-40 μM) and **(B)** Ni^{2+} (0-40 μM) in MeOH solution at 25 °C. **(C)** The ratio of absorbance at 525 nm of compound **DTEP** (10 μM) at photostationary state without and with various metal ions (40 μM) in MeOH solution at 25 °C. Inset: the corresponding photographic images of compound **DTEP** in the photostationary state with various metal ions.

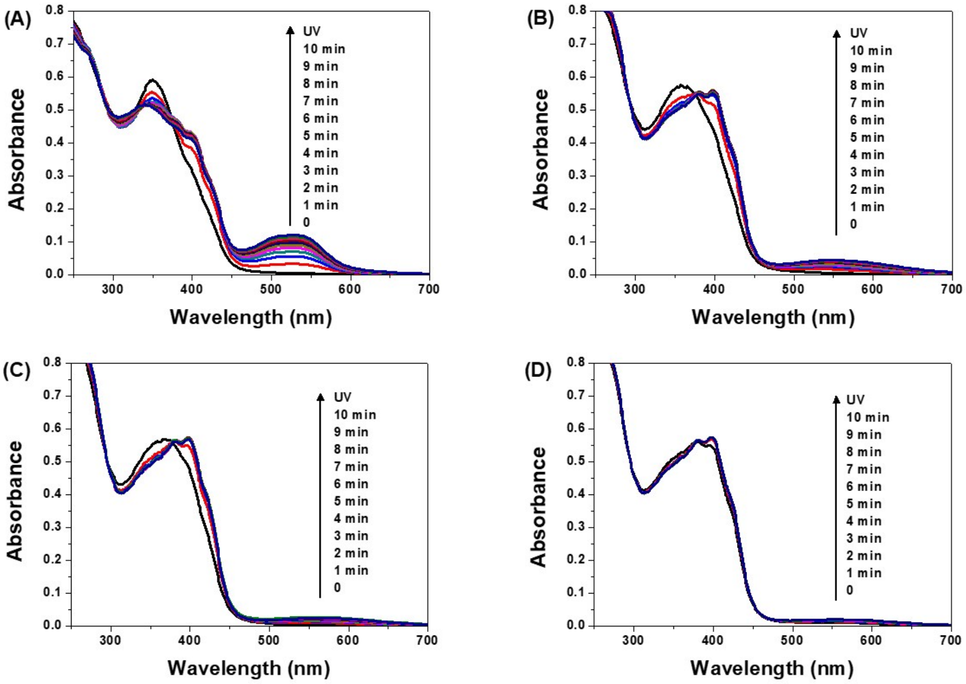

Figure S8. UV-vis absorption spectral changes of compound **DTEP** (10 μM) with different amounts of Cu^{2+} upon irradiation with 365 nm light in MeOH solution at 25 ℃: **(A)** 1 equiv.; **(B)** 2 equiv.; **(C)** 3 equiv. and **(D)** 4 equiv.

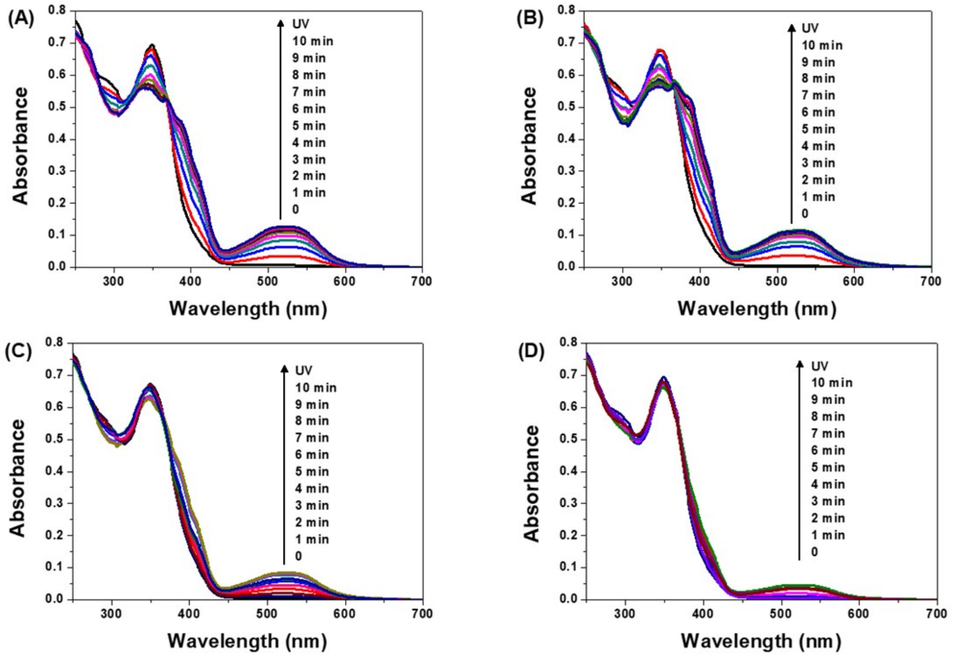

Figure S9. UV-vis absorption spectral changes of compound **DTEP** (10 μM) with different amounts of Ni^{2+} upon irradiation with 365 nm light in MeOH solution at 25 ℃: **(A)** 1 equiv.; **(B)** 2 equiv.; **(C)** 3 equiv. and **(D)** 4 equiv.

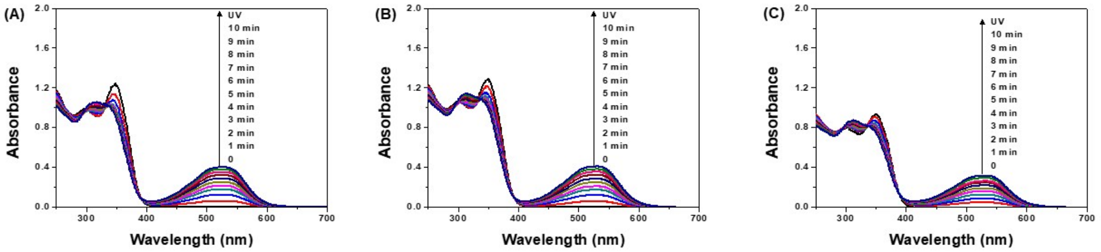

Figure S10. UV-vis absorption spectral changes of compound **DTEP** (10 μM) with different proportion of water upon irradiation with 365 nm light in MeOH-H_2O solution at 25 ℃: **(A)** 9: 1, v/v.; **(B)** 7: 3, v/v and **(C)** 5: 5, v/v.

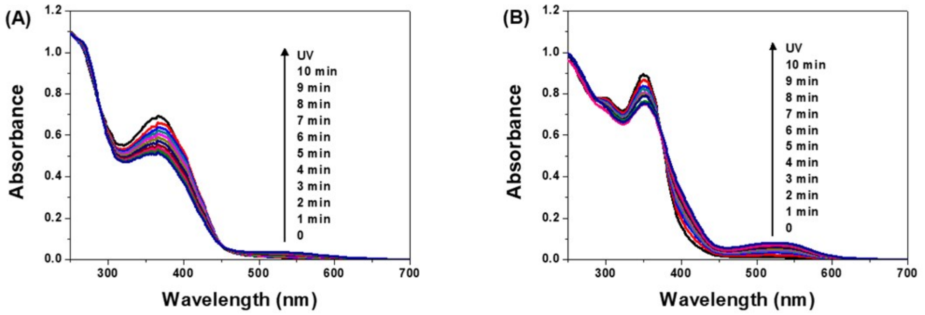

Figure S11. UV-vis absorption spectral changes of compound **DTEP** (10 μM) with **(A)** Cu^{2+} (40 μM) and **(B)** Ni^{2+} (40 μM) upon irradiation with 365 nm light in MeOH-H_2O (5: 5, v/v) solution at 25 ℃.

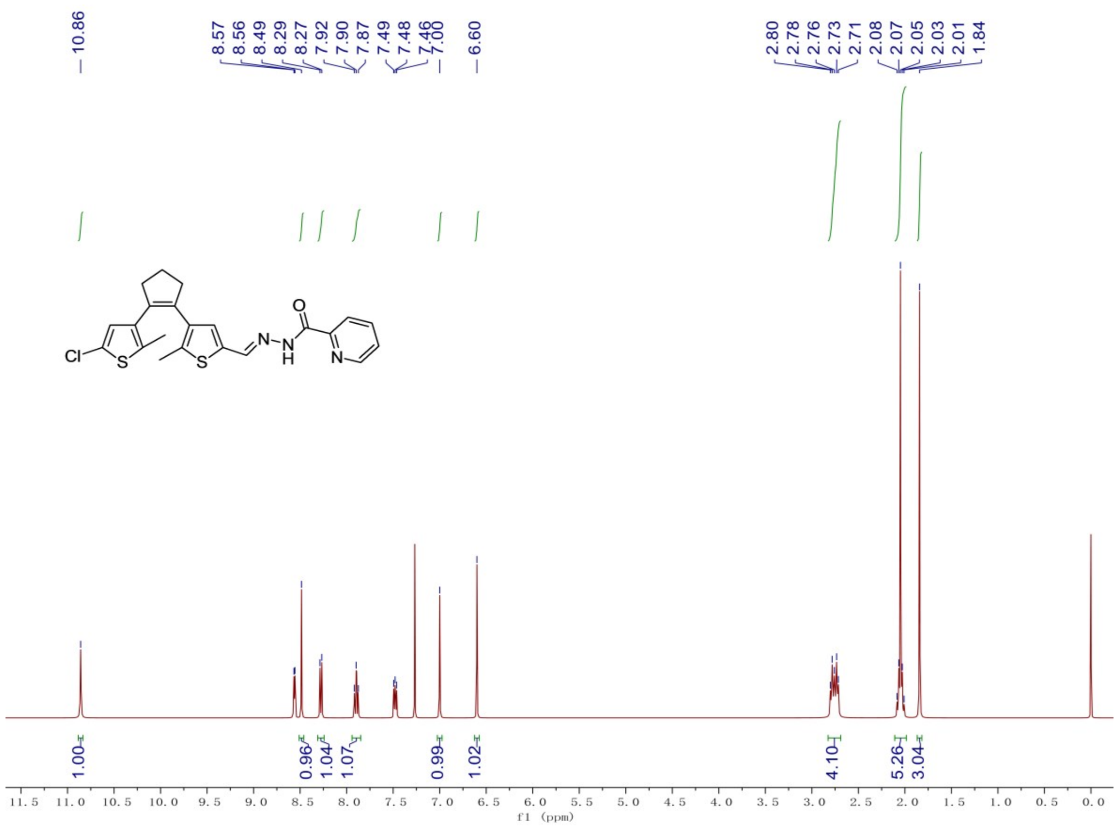

Figure S12. ^{1}H NMR ($CDCl_3$, 400 MHz) spectrum of compound **DTEP**.

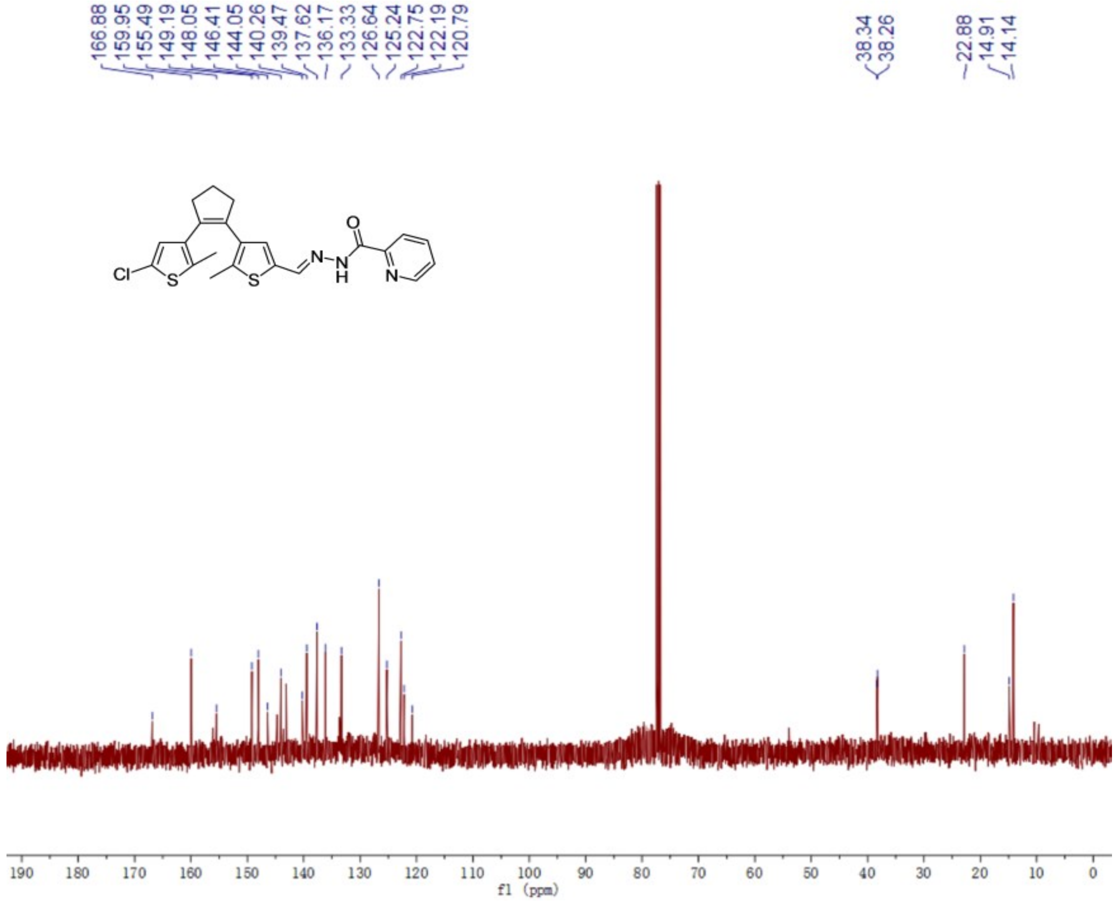

Figure S13. ^{13}C NMR ($CDCl_3$, 100 MHz) spectrum of compound **DTEP**.

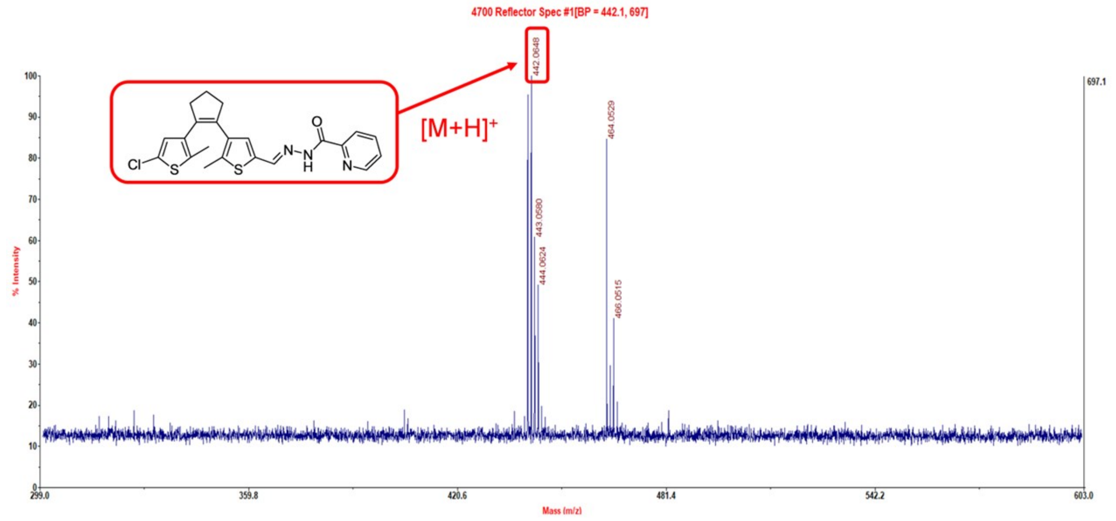

Figure S14. MS (MALDI-TOF) of compound **DTEP**.
